# PLZF Controls the Expression of a Limited Number of Genes Essential for NKT Cell Function

**DOI:** 10.3389/fimmu.2012.00374

**Published:** 2012-12-21

**Authors:** Michael Gleimer, Harald von Boehmer, Taras Kreslavsky

**Affiliations:** ^1^Laboratory of Lymphocyte Biology, Dana-Farber Cancer Institute, Harvard Medical SchoolBoston, MA, USA; ^2^Department of Microbiology and Immunobiology, Harvard Medical SchoolBoston, MA, USA

**Keywords:** NKT, PLZF, c-Maf, Id2, CXCR6, ICOS, IL12R, IL18R

## Abstract

Natural killer (NKT) T cells exhibit tissue distribution, surface phenotype, and functional responses that are strikingly different from those of conventional T cells. The transcription factor PLZF is responsible for most of these properties, as its ectopic expression in conventional T cells is sufficient to confer to them an NKT-like phenotype. The molecular program downstream of PLZF, however, is largely unexplored. Here we report that PLZF regulates the expression of a surprisingly small set of genes, many with known immune functions. This includes several established components of the NKT cell developmental program. Expression of the transcriptional regulators *Id2*, previously shown to be required for iNKT cell survival in the liver and *c-Maf*, which shapes the NKT cytokine profile, was compromised in PLZF-deficient cells. Ectopic expression of *c-Maf* complemented the cells’ defect in producing IL-4 and IL-10. PLZF also induced a program of cell surface receptors which shape the NKT cell’s response to external stimuli, including the costimulatory receptor *ICOS* and the cytokine receptors *IL12rb1* and *IL18r1*. As an ensemble, the known functions of the molecules whose expression is affected by PLZF explain many defects observed in *PLZF^−/−^* NKT cells.

## Introduction

Multiple T cell lineages develop in the thymus from common progenitors. Most T lymphocytes – so called conventional T cells – exit the thymus in the “naïve” state. After activation by cognate antigen they take days to differentiate into effector cells capable of cytokine secretion and cytotoxic responses. Several T cell subsets, however, acquire effector functions already in the thymus – presumably because they encounter TCR ligands but escape deletion (Baldwin et al., 2004). Such cells exhibit rapid effector responses resembling those of innate immune cells (e.g., natural killer; NK cells), and are thus called “innate-like” T cells. They are believed to represent the first line of defense against pathogens, and to provide cytokines that regulate subsequent immune responses by conventional T cells.

Exactly how innate-like T cells acquire their peculiar properties is poorly understood. Perhaps the best studied subset of innate T lymphocytes are the invariant natural killer T (iNKT) cells. In mice, they express an αβ T cell receptor (TCR) that consists of the invariant Vα14-Jα18 TCRα chain coupled with TCRβ chains of limited diversity, and recognize lipid antigens in the context of the MHC class I-like molecule CD1d. Many iNKT cells express cell surface markers typical of NK cells, including NK1.1, but can also express the CD4 coreceptor, and, like other innate-like T cells, exhibit in the absence of deliberate activation a surface phenotype characteristic of activated conventional T cells. Upon activation, iNKT cells rapidly secrete a unique spectrum of cytokines. Specifically, they can co-produce IFNγ and IL-4 – i.e., cytokines secreted by discrete subsets of conventional T cells – Th1 and Th2 cells, respectively. Another key feature differentiating NKT cells from conventional T cells is their ability to produce cytokines in response to proinflammatory stimuli in the absence of foreign antigen – a property that is dependent on signaling by IL-12 (Brigl et al., 2003, 2011), IL-18 (Nagarajan and Kronenberg, 2007; Velazquez et al., 2008), or type I interferon (Paget et al., 2007) receptors. Unlike naïve conventional T cells, which home to spleen and lymph nodes, iNKT cells accumulate in the liver, but are also present in the spleen and, to a lesser extent, lymph nodes.

A subset of γδ T cells that express the Vγ1Vδ6.3/Vδ6.4 TCR exhibits properties resembling those of iNKT cells (Azuara et al., 1997; Lees et al., 2001). Unlike most other γδ T cells, they often express NK1.1 and CD4, home to the liver, can co-produce IL-4 and IFNγ, and depend on the same transcriptional program as iNKT cells (Azuara et al., 1997; Lees et al., 2001; Alonzo et al., 2009; Kreslavsky et al., 2009; see below). Thus, they are often referred to as γδNKT cells.

Significant advances were made in understanding the transcriptional network that regulates NKT cell development. A plethora of transcription regulators including, but not restricted to GATA3, T-bet, ThPOK, Id2, Runx1, Tox, c-Myc, Egr2, and c-Maf were implicated in iNKT cell development, function, and/or homeostasis (reviewed in D’Cruz et al., 2010; Godfrey et al., 2010). Recently it was reported that the transcription factor PLZF (promyelocytic leukemia zinc finger), previously implicated in limb patterning (Barna et al., 2000) and spermatogonial stem cell self-renewal (Buaas et al., 2004), was required for iNKT cell development. In the absence of PLZF, CD1d-restricted iNKT cells were present, but their numbers were severely reduced in the thymus, spleen, and liver (Kovalovsky et al., 2008; Savage et al., 2008). iNKT cells in PLZF-deficient mice resembled conventional T cells because of their reduced expression of activation markers and diminished ability for rapid cytokine production (Kovalovsky et al., 2008; Savage et al., 2008). Similar changes were documented for PLZF-deficient γδNKT cells (Alonzo et al., 2009; Kreslavsky et al., 2009). The observation that PLZF could be induced by TCR crosslinking in polyclonal TCRγδ^+^ thymocytes (Kreslavsky et al., 2009) suggested that PLZF induction may require the encounter of TCR ligands in the thymus. Indeed, a novel reporter mouse suggests that NKT cells undergo strong TCR signaling at an early stage of their development (Moran et al., 2011); consistently, Egr2 downstream of the TCR has been shown to activate PLZF expression (Seiler et al., 2012). Strikingly, transgenic PLZF expression in conventional T cells was sufficient to confer to them many NKT cell properties (Raberger et al., 2008; Savage et al., 2008, 2011; Kovalovsky et al., 2010). Thus PLZF appears both necessary and sufficient for the acquisition of many NKT cell-like properties by a conventional T cell – and seems to be absolutely unique in this respect.

PLZF expression by NKT and NKT-like cells also has a far-reaching influence on other immune cells. Mice deficient in *Itk* (Felices et al., 2009; Qi et al., 2009), *Id3* (Lauritsen et al., 2009; Ueda-Hayakawa et al., 2009; Verykokakis et al., 2010a), and *KLF2* (Odumade et al., 2010), as well as mice with mutated *SLP-76* (Alonzo et al., 2009) and mice transgenic for Dok-1 (Besin et al., 2012) have increased numbers of PLZF-expressing cells – a phenomenon yet to be explained. IL-4 secretion by these PLZF-expressing cells leads to acquisition of innate-like features by CD8 T cells (Verykokakis et al., 2010b; Weinreich et al., 2010; Gordon et al., 2011). The increased frequency of innate-like CD8 T cells in wt BALB/c mice likewise depends on IL-4 produced by NKT cells (Verykokakis et al., 2010b; Weinreich et al., 2010; Lai et al., 2011).

Even though the defects in the NKT cell compartment of PLZF-deficient mice are well-defined, little is known about the molecular program downstream from PLZF. Here we demonstrate that PLZF regulates the expression of a very restricted set of genes, many of which have known immune function. This includes known regulators of NKT cell development, function, and homeostasis: the transcription factor *c-Maf*, required for IL-4 production (Kim et al., 1999); the inhibitor of E protein signaling *Id2*, required for survival and accumulation of NKT cells in the liver (Monticelli et al., 2009); the receptor ICOS (inducible T cell costimulator), required for accumulation of NKT cells in thymus, spleen, and liver, as well as for cytokine responses (Akbari et al., 2008; Chung et al., 2008; Watanabe et al., 2010); and the cytokine receptors *IL12rb1* and *IL18r1*, required for effector responses in the absence of foreign antigen (Brigl et al., 2003, 2011; Nagarajan and Kronenberg, 2007; Velazquez et al., 2008). The known, NKT cell-specific functions of these molecules can explain to a large extent the abnormal NKT cell phenotype of PLZF-deficient animals with respect to tissue distribution, secretion of and responsiveness to cytokines.

## Materials and Methods

### Mice

Luxoid mice that bear a point mutation in the PLZF gene (Buaas et al., 2004) and phenocopy the PLZF knockout in the NKT compartment (Kovalovsky et al., 2008; Savage et al., 2008; backcrossed for more than 35 generations to C57BL/6) were obtained from the Jackson Laboratory. C57BL/6 and CD45.1 C57BL/6 mice were obtained from the Jackson Laboratory and Taconic, respectively. Rag1^−/−^ Vγ1Vδ6.4 TCR transgenic mice were described previously (Kreslavsky et al., 2009). All mice were bred and maintained in the animal facility at the Dana-Farber Cancer Institute (DFCI). All animal procedures were done in compliance with the guidelines of the DFCI Animal Resources Facility, which operates under regulatory requirements of the U.S. Department of Agriculture and the Association for Assessment and Accreditation of Laboratory Animal Care.

### Flow cytometry and cell sorting

mAbs specific for CD4 (RM4-5), CD8a (53-6.7), TCRβ (H57-597), TCRγδ (GL3), NK1.1 (PK136), CD24 (M1/69), CD44 (IM7), CD45.1 (A20), CD45.2 (104), ICOS (C398.4A and 15F9), Vδ6.3 (8F4H7B7), IL12Rβ1 (114), IL18R1 (BG/IL18RA), IFNγ (XMG1.2), CXCR6 (221002), IL-10 (JES5-16E3), and IL-4 (BVD6-24G2) were purchased from BD Biosciences, eBioscience, R&D Biosystems, or Biolegend and were used as fluorescein isothiocyanate (FITC), phycoerythrin (PE), peridinin chlorophyll protein (PerCP), PerCP-Cy5.5, PE-Cy7, allophycocyanin (APC), APC-Cy7, Alexa-Fluor^®^ 647, or Pacific Blue conjugates. PLZF antibody (D-9) was purchased from Santa Cruz Biotechnology and conjugated to Alexa-Fluor 647 using a kit from Invitrogen. For intracellular PLZF detection cells were fixed and permeabilized using the eBioscience Foxp3 Staining Buffer Set. PE and Pacific Blue labeled PBS-57-loaded CD1d tetramers were obtained from the NIH Tetramer Facility. For cytokine production cells were stimulated with 50 ng/mL phorbol 12-myristate 13-acetate (PMA) and 500 ng/mL ionomycin for 5 h with brefeldin A (3 μg/mL) added after 1 h of incubation, or left unstimulated. Cells were then stained for surface markers, fixed, permeabilized using the BD Cytofix/Cytoperm kit and stained for intracellular cytokines. Flow cytometry and cell sorting was performed on a FACSAria (BD Biosciences) cell sorter. Data were analyzed using FlowJo (Treestar).

### Retroviral infections

Full-length protein-coding c-Maf (Ensembl transcript ID ENSMUST00000109104) cDNA was cloned into pMIG retroviral vector (Addgene). Retrovirus-containing supernatants were generated by transient co-transfection of 293T cells with pMIG based constructs and packaging vector pCL-Eco (Addgene). Supernatants were harvested on day 4 and concentrated on centrifugal filtration devices (100 kDa cutoff; Millipore). Cells were spin-infected (1.5 h, 500 g) on plates coated with RetroNectin (Takara) in the presence of 8 μg/mL polybrene (Sigma).

### NKT cell cultures

CD1d-PBS57-Tet^+^ cells were sorted from WT and luxoid mice and cultured for 2 days in the presence of 10 ng/mL IL-7 (R&D Systems) and 100 ng/mL IL-15 (Peprotech). Cells were then spin-infected with c-Maf-encoding or control retroviruses as described above in the presence of IL-7 and IL-15. Two days after infection cells were washed, stimulated with PMA/ionomycin and stained for intracellular cytokines as described above. For cytokine stimulation, 10 ng/mL IL-12 and/or 100 ng/mL IL-18 (Peprotech) or plates coated with 10 μg/mL CD3e antibody (145-2C11) were used. IFNγ in culture supernatants was measured using the mouse IFNγ OptEIA kit (BD Pharmingen).

### Generation of mixed bone marrow chimeras

Bone marrow cells from CD45.1 C57BL/6 and CD45.2 luxoid mice were stained with CD4, CD8α, TCRβ, TCRγδ, and NK1.1 biotinylated antibodies followed by incubation with streptavidin-conjugated magnetic beads (Invitrogen) and magnetic bead depletion of T and NK cells. CD45.1 C57BL/six recipients were the subject of lethal irradiation (1000 rads) using a γ-cell 40 irradiator with a cesium source. Mixed (1:1) T cell- and NK-cell-depleted wt and PLZF^lu/lu^ bone marrow cells (4–8 × 10^6^) were intra-orbitally injected into the recipient mice. Chimeras were used in experiments 5 weeks or more after the bm transfer.

### Microarrays

**Figure 1:**
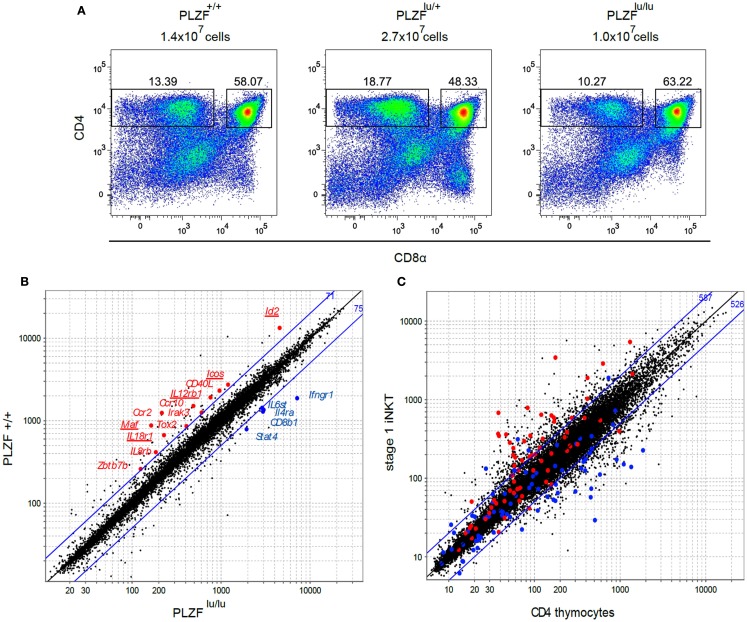
**Cell surface phenotype and gene expression profiles of thymocytes from PLZF sufficient and PLZF-deficient TCR transgenic mice**. **(A)** Thymocytes from Rag1^−/−^Vγ1Vδ6.4 TCR transgenic animals on PLZF^+/+^, PLZF^lu/+^, and PLZF^lu/lu^ backgrounds were stained with antibodies against CD4 and CD8α and analyzed by FACS. Absolute numbers of thymocytes are indicated. Representative FACS plots from one of two independent experiments are shown. **(B)** Expression profiles of PLZF^+/+^ versus PLZF^lu/lu^ double-sorted CD4^+^CD8^−^ thymocytes from Rag1^−/−^Vγ1Vδ6.4 TCR transgenic animals were compared. Expression values of PLZF^lu/lu^ (*X* axis) against PLZF^+/+^ (*Y* axis) cells are plotted. Blue lines indicate twofold difference thresholds in expression between PLZF^+/+^ and PLZF^lu/lu^ thymocytes. Numbers in blue indicate the number of probe sets above the upper line and below the lower line. Some of the immunologically relevant genes are highlighted and labeled. **(C**) Expression values of conventional CD4 thymocytes (*X* axis) against stage 1 NKT cells (*Y* axis) from Immgen database are plotted. Genes positively regulated by PLZF above the twofold threshold in (**B**) are highlighted in red, those regulated negatively – in blue. Blue lines indicate twofold difference thresholds in expression. Numbers in blue indicate the number of probe sets above the upper line and below the lower line.

CD4^+^CD8α^−^ thymocytes from Vγ1Vδ6.4 transgenic mice on Rag1^−/−^ PLZF^lu/lu^, PLZF^lu/+^, and PLZF^+/+^ backgrounds were double-sorted, with the second sort directly into TRIzol reagent (Invitrogen). Total RNA was prepared and submitted to the microarray core facility at the DFCI. There labeled cDNA was prepared and hybridized to Affymetrix Mouse Gene 1.0 ST Arrays. Data processing and analysis was performed using the GenePattern v3.21 package (http://genepattern.broadinstitute.org) using ExpressionFileCreator, MultiplotPreprocess, and Multiplot modules. Probe sets corresponding to normalization controls were filtered out prior to the analysis. Data have been submitted to GEO (accession number GSE42168). For comparison shown in Figure 1C dataset from Immgen database (Heng and Painter, 2008) was used (GEO accession number GSE15907).

### Quantitative PCR

Indicated populations were double-sorted, with the second sort directly into TRIzol reagent (Invitrogen). Total RNA was prepared and cDNA was synthesized using Superscript III reverse transcriptase (Invitrogen) according to the manufacturer’s recommendations. Real-time RT-PCR was performed on an ABI PRISM thermal cycler (Applied Biosystems) using TaqMan PCR mastermix (Applied Biosystems). Mm02581355_s1 (Maf), Mm00711781_m1 (Id2), and Mm99999915_g1 (GAPDH) TaqMan Gene Expression Assays (Applied Biosystems) were used.

## Results

### PLZF regulates a focused program of factors important for NKT cell function

To dissect PLZF-dependent regulation of NKT cells at the molecular level we aimed to compare gene expression profiles of cells from WT and PLZF-deficient luxoid (*PLZF^lu/lu^*) mice (Savage et al., 2008). However, both iNKT (Kovalovsky et al., 2008; Savage et al., 2008) and γδNKT (Alonzo et al., 2009) cell numbers are very low in the *PLZF*-deficient animals, and the residual cells may be the product of selection of an NKT subpopulation, and therefore may not be directly comparable to wt counterparts (Savage et al., 2008). To circumvent these problems we crossed luxoid animals to mice transgenic for a γδNKT TCR (Vγ1Vδ6.4), which was previously shown to generate large numbers of PLZF-expressing T cells (Kreslavsky et al., 2009). Since the phenotypes of γδNKT and iNKT cells were very similar in *PLZF^lu/lu^* animals, the molecular program downstream of PLZF in these subsets should overlap to a great extent.

The gross thymic phenotype of *Rag1^−/−^*γδ*TCR* transgenic *PLZF^lu/lu^, PLZF^lu/^+*, and *PLZF*^+*/*+^ animals – including thymic cellularity and the frequency of CD4^−^CD8^−^, CD4^+^CD8^+^, and CD4^+^CD8^−^ cells – was very similar (Figure 1A). From this we concluded that there was no evidence for selection of a subpopulation in these mice, and proceeded to compare γδNKT cells from TCR transgenic *PLZF*-sufficient and *PLZF*-deficient mice. We previously demonstrated that PLZF in the γδTCR transgenic mice was expressed by the majority (78.8 ± 3.9%) of CD4^+^CD8^−^ thymocytes, and that PLZF upregulation required the expression of the TCR transgene (Kreslavsky et al., 2009). The CD4^+^ phenotype of γδNKT cells was not surprising, since a large fraction of both αβ and γδNKT cells in wt mice express CD4 (Azuara et al., 1997).

Gene expression profiles of CD4^+^CD8^−^ thymocytes from *Rag1^−/−^* *V*γ*1V*δ*6.4* transgenic mice on *PLZF^lu/lu^, PLZF^lu/^+*, or *PLZF*^+*/*+^ backgrounds were compared using Affymetrix Mouse Gene 1.0 ST chips. Since without PLZF NKT cells resemble naïve T cells in their surface phenotype and functional properties (Kovalovsky et al., 2008; Savage et al., 2008) and since ectopic PLZF expression is sufficient to confer memory/effector properties to conventional T cells in the absence of agonist TCR signaling (Raberger et al., 2008; Kovalovsky et al., 2010; Savage et al., 2011), we expected to find differences in expression of many genes, comparable to the differences between conventional T cells and NKT cells. Strikingly, however only 146 probe sets exhibited more than a twofold difference between *PLZF^lu/lu^* and *PLZF*^+*/*+^ thymocytes, compared to over a thousand probe sets between wt CD4SP thymocytes and wt stage 1 NKTs (Figures 1B,C). A number of genes with known immune function were expressed at higher levels in the presence of PLZF. These include genes for chemokine receptors *Ccr2, Ccr10*, and *Il8rb*, cytokine receptors *Il12rb1* and *Il18r1*, transcription regulators *Id2, Zbtb7b* (encodes ThPOK), and *Maf* (encodes c-Maf), as well as costimulatory molecules Icos and Cd40lg (Figure 1B; Table S1 in Supplementary Material). Among genes that were downregulated in the presence of PLZF were *Il4ra, Ifngr1, Stat4, Il6st*, and *Cd8b1* (Figure 1B and Table S2 in Supplementary Material). Many PLZF-regulated genes from our analysis were differentially expressed between iNKT and CD4+ thymocytes (Figure 1C), consistent with our gene set representing the NKT molecular program. Since Id2 (Monticelli et al., 2009), c-Maf (Kim et al., 1999), as well as ICOS (Akbari et al., 2008; Chung et al., 2008; Watanabe et al., 2010), IL12R (Brigl et al., 2003, 2011), and IL18R (Nagarajan and Kronenberg, 2007; Velazquez et al., 2008) signaling pathways had previously been implicated in NKT cell development, homeostasis and/or function, we proceeded to validate these targets in iNKT and γδNKT cells from wt and PLZF-deficient mice that did not express TCR transgenes.

### PLZF/c-Maf axis controls IL-4 and IL-10 production

**Figure 2:**
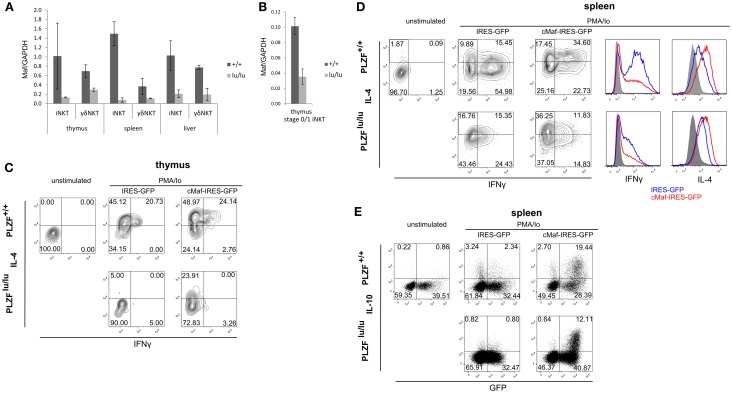
**PLZF regulates the expression of c-Maf**. Expression of Maf was analyzed by TaqMan qPCR in sorted total thymic, splenic, and liver iNKT (TCRβ^+^CD1d-Tet^+^) and γδNKT (TCRγδ^+^Vδ6.3^+^) cells **(A)** or in stage 0/stage 1 iNKT cells (CD44^−^NK1.1^−^TCRβ^+^CD1d-Tet^+^) **(B)** from mixed bm chimeras. Mean relative c-Maf expression normalized against GAPDH expression is shown. Error bars represent SD (individual mice). Statistical significance is indicated where reached. iNKT cell were sorted from thymi **(C)** and spleens **(D,E)** of WT or PLZF^lu/lu^ mice, cultured for 2 days in the presence of IL-7 and IL-15, infected with IRES-GFP or c-Maf-IRES-GFP retroviruses and stimulated with PMA/Ionomycin 2 days after infection. Expression of IFNγ and IL-4 in GFP^+^ cells **(C,D)** as well as IL-10 and GFP in all NKT cells **(E)** is shown. Representative FACS plots from one of two **(C)** and one of three **(D,E)** experiments are shown. Histogram overlays comparing cytokine production by wt and lu/lu cells in response to c-Maf expression are shown for **(D)**.

Strong reduction in IL-4 and IL-10 production by NKT cells is a characteristic feature of the PLZF^−/−^ phenotype (Kovalovsky et al., 2008; Savage et al., 2008). The transcription factor c-Maf is required for IL-4 production by Th2 and NKT cells (Ho et al., 1996; Kim et al., 1999), and was shown to regulate IL-10 expression in Th17 and Tr1 cells (Xu et al., 2009b; Apetoh et al., 2010). We thus measured *c-Maf* expression in TCR non-transgenic iNKT and γδNKT cells. To exclude non-cell autonomous effects of *PLZF* deficiency, this was done in mixed bm chimeras. *PLZF^lu/lu^* iNKT and γδNKT cells exhibited a significant reduction in *c-Maf* expression (Figure 2A). The level of *c-Maf* was close to that in conventional CD4 thymocytes (Figure A2A in Appendix).

It was suggested that iNKT cells in *PLZF*-deficient mice did not progress beyond stage 1 (NK1.1^−^CD44^−^) in their development (Savage et al., 2008) and thus the observed differences in gene expression may result from this developmental block (arguments for and against this block are discussed later). Thus we compared *c-Maf* expression in CD1d tetramer binding NK1.1^−^CD44^−^ thymocytes (includes stage 0 and stage 1 iNKT cells). Although the overall level of *c-Maf* expression was lower in these cells when compared to total iNKT cells, the reduction was obvious (Figure 2B). Thus PLZF is required for the upregulation of *c-Maf* before the putative stage 1 developmental block.

We next tested whether *c-Maf* expression could complement cytokine production defects in *PLZF*-deficient NKT cells. Due to the extremely low number of γδNKT cells in *PLZF^lu/lu^* animals we focused on iNKT cells for this experiment. As premature expression of *c-Maf* can lead to a block in T cell development (Morito et al., 2006) we sought to restore *c-Maf* expression in mature iNKT cells. To this end, sorted iNKT cells from wt and *PLZF^lu/lu^* animals were infected with c-Maf-IRES-GFP or control IRES-GFP retroviruses. Cells were then stimulated with PMA/ionomycin, and production of IL-4, IFNγ, and IL-10 was assessed. In thymic iNKT cells, ectopic *c-Maf* expression rescued IL-4 production by PLZF-deficient cells and further increased the level of IL-4 per cell in wt iNKT cells (Figure 2C). The same trend was observed in splenic iNKT cells (Figure 2D), although some IL-4 was already produced by stimulated *PLZF^lu/lu^* splenic iNKT cells – presumably because of IL-7 and IL-15 in culture. Likewise, ectopic *c-Maf* expression rescued IL-10 production by PLZF-deficient splenic iNKT cells, and further enhanced IL-10 production by their wt counterparts (Figure 2E). *c-Maf* overexpression somewhat reduced the production of IFNγ (Figure 2D), consistently with previous reports for conventional T cells (Ho et al., 1998). However, the frequency of IFNγ/IL-4 dual producers – a population most affected by PLZF deficiency – was reproducibly increased by ectopic *c-Maf* expression in splenic wt iNKT cells.

Thus the expression of *c-Maf*, a transcription factor that regulates cytokine production, is induced by PLZF and its ectopic expression is sufficient to rescue IL-4 and IL-10 production by *PLZF*-deficient iNKT cells.

### PLZF is required for appropriate expression of Id2 in NKT cells

In *PLZF*-deficient mice, the reduction in the NKT compartment in the liver is more dramatic than that in the thymus and spleen (Savage et al., 2008), suggesting that the former is not simply a result of the latter.

Mice deficient for an inhibitor of E protein signaling – *Id2* – were recently demonstrated to have about 10-fold lower numbers of NKT cells in the liver – but not in the spleen or thymus (Monticelli et al., 2009). The phenotype was attributed to cell survival. A recent report suggests that *Id2* is a direct PLZF target in myeloid cells (Doulatov et al., 2009). Thus we tested whether *Id2* expression depends on PLZF in NKT cells from mice that did not express a TCR transgene.

**Figure 3:**
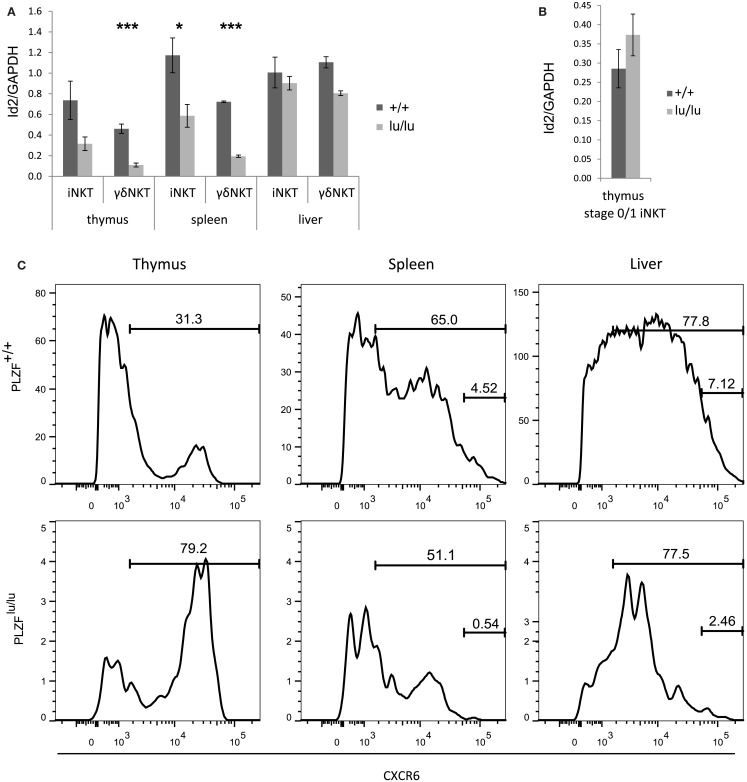
**PLZF regulates the expression of Id2**. Expression of Id2 was analyzed by TaqMan qPCR in sorted total thymic, splenic, and liver iNKT (TCRβ^+^CD1d-Tet^+^) and γδNKT (TCRγδ^+^Vδ6.3^+^) cells **(A)** or in stage 0/1 iNKT cells (CD44-NK1.1-TCRβ^+^CD1d-Tet^+^) **(B)** from mixed bm chimeras. Mean relative Id2 expression normalized against GAPDH expression is shown. Error bars represent SD (individual mice). Statistical significance is shown where reached. **(C)** CXCR6 expression was analyzed on wt (CD45.1) or PLZF^lu/lu^ (CD45.2) iNKT (TCRβ^+^CD1d-Tet^+^) cells from the thymus, spleen, and liver of bm chimeric mice. Percent CXCR^+^ and CXCR^hi^ are indicated.

PLZF-deficient thymic and splenic iNKT and γδNKT cells (Figure 3A) from mixed bone marrow chimeras exhibited a twofold to threefold reduction in *Id2* transcripts when compared to wt cells. Both *PLZF^lu/lu^* and wt stage 0/1 NKT cells exhibited comparably low levels of *Id2* expression (Figure 3B). Interestingly, the residual *PLZF*-deficient NKT cells from the liver exhibited a normal level of *Id2* expression. As *Id2* was shown to control survival of hepatic NKT cells, this result may indicate selection for cells with a normal level of expression.

Id2^−/−^ NKT cells also show decreased expression of CXCR6, a receptor important for homing and accumulation of iNKT cells in the liver (Monticelli et al., 2009). Consistently, PLZF-deficient iNKT cells in spleen and liver had a much reduced CXCR6^hi^ population (Figure 3C). Some CXCR6^hi^ cells remained in the thymus, however, perhaps explaining why CXCR6 was not differentially expressed on our microarray.

### ICOS expression in NKT cells is affected by PLZF

**Figure 4:**
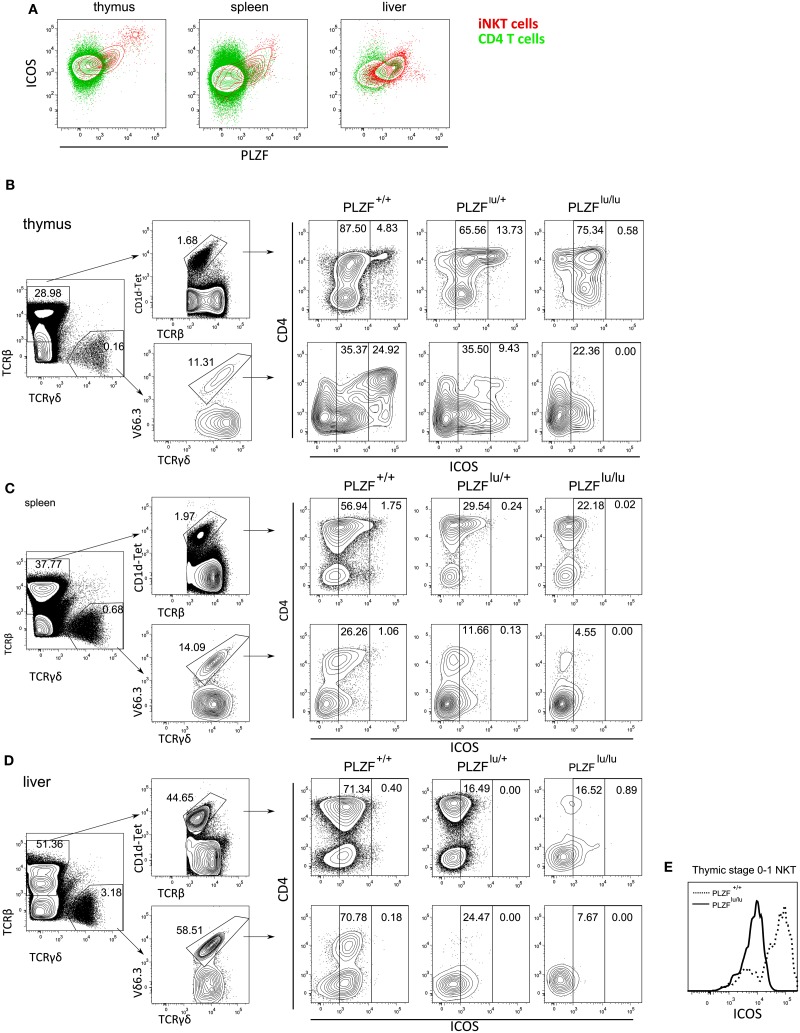
**PLZF regulates the expression of ICOS**. Cells from thymus, spleen and liver were stained with CD1d-PBS-57 tetramers and antibodies against ICOS and CD4, fixed, permeabilized, stained with anti-PLZF and analyzed by FACS **(A)**. Results are depicted as an overlay of ICOS/PLZF plots for CD4^+^CD1d^−^Tet^−^ (green) and CD1d-Tet^+^ cells (red). Cells from thymi **(B)**, spleens **(C)**, and livers **(D)** of PLZF^lu/lu^, PLZF^lu/+^, and PLZF^+/+^ mice were stained with PBS-57-loaded CD1d tetramers and antibodies against TCRβ, TCRγδ, Vδ6.3, CD4, and ICOS, and analyzed by FACS. Gating scheme for WT cells is shown (left). Expression of CD4 and ICOS on TCRβ^+^CD1d-tet^+^ and TCRγδ^+^Vδ6.3^+^ cells is shown for mice with each genotype (right). Gates are set on ICOS^int^ and ICOS^hi^ cells. Expression of ICOS on thymic stage 0/stage 1 iNKT cells (CD44^−^NK1.1^−^TCRβ^+^CD1d-Tet^+^) is shown. **(E)** Representative FACS plots from one of three independent experiments are shown.

Many features of the *PLZF^−/−^* phenotype – such as reduced NKT cell numbers in spleens and livers, a defect in production of cytokines – including, but not restricted to IFNγ and IL-4 – were also reported for mice deficient in the costimulatory molecule ICOS or for its ligand (Akbari et al., 2008; Chung et al., 2008; Watanabe et al., 2010), albeit these traits were less pronounced in *ICOS^−/−^* and *ICOSL^−/−^* mice than in *PLZF^−/−^* animals. Like in the case of PLZF deficiency, this was at least in part a developmental defect, rather than merely a defect in activation due to the lack of costimulation, as a partial block in iNKT cell differentiation was observed (Chung et al., 2008). Interestingly, ICOS and PLZF exhibited correlated expression in iNKT cells (Figure 4A), suggesting that PLZF was a limiting factor in the regulation of ICOS expression in NKT cells. In the thymus a fraction of PLZF^hi^ NKT cells expressed very high levels of ICOS. PLZF^hi^ cells correspond to immature stages 1 and 2 of NKT cell development (Kovalovsky et al., 2008) – and thus NKT cells progress through a PLZF^hi^ICOS^hi^ stage early in their differentiation.

**Figure A1:**
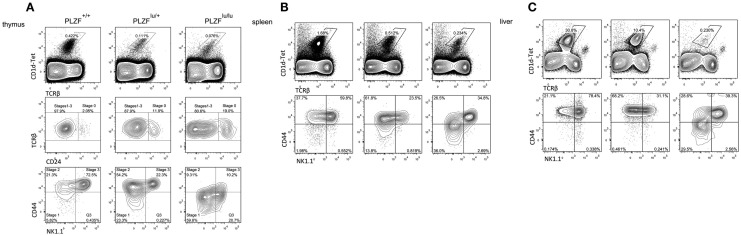
**Surface phenotype of iNKT cells from PLZF^+/+^, PLZFl^u/+^ and PLZF^lu/lu^ mice**. Cells from thymi **(A)**, spleens **(B)**, and livers **(C)** of mice with indicated genotype were isolated and stained with PBS-57-loaded CD1d tetramers and antibodies against TCRß, CD24, CD44 and NK1.1.

As the phenotypes of *ICOS^−/−^* and *ICOSL^−/−^* animals were reminiscent of the *PLZF* knockout, we next measured the expression of ICOS in *PLZF*-deficient, *PLZF*-heterozygous, and wt mice. ICOS^hi^ cells, which were readily detectable among both iNKT and γδNKT WT thymocytes, predominantly within the CD4^+^ population, were completely absent in the *PLZF^lu/lu^* thymi. ICOS^int^ iNKT and γδNKT cells were present at a comparable frequency in the thymus (Figure 4B). *PLZF^lu/+^* iNKT thymocytes contained an abnormally high percentage of ICOS^hi^ cells (Figure 4B) – a finding explained by the fact that in these animals a large number of cells appear blocked at the ICOS^hi^ stage 2 (Figure A1A in Appendix). In the periphery (Figures 4C,D) the ICOS^hi^ population was not detectable, consistent with the fact that they represent immature cells. However, expression of ICOS on peripheral iNKT and γδNKT cells was dramatically reduced in *PLZF*-deficient cells, resulting in a strong reduction in ICOS^int^ cells in the spleen (Figure 3C) and liver (Figure 4D). Notably, the regulation of ICOS by PLZF was evident even in early thymic stage 0/1 iNKT cells (Figure 4E), suggesting that the effect was independent of any possible developmental block.

### PLZF programs NKT cells to respond to a distinct set of cytokines

**Figure 5:**
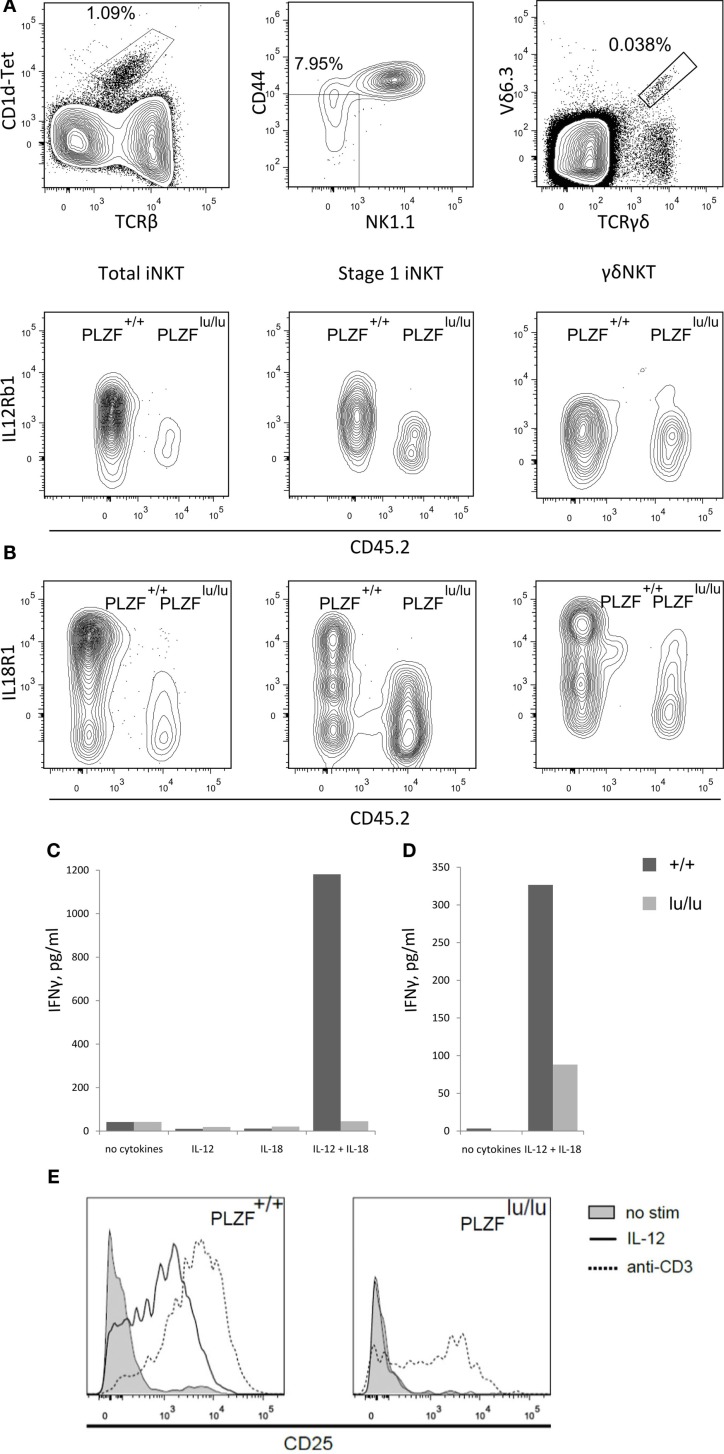
**PLZF regulates the expression of receptors for IL12 and IL18**. Thymocytes from mixed chimeras with wt (CD45.1) and PLZF^lu/lu^ (CD45.2) bm were stained with PBS-57-loaded CD1d tetramers and antibodies against the indicated surface markers. Expression of IL12Rb1 **(A)** and IL18R1 **(B)** on total iNKT cells (TCRβ^+^CD1d-Tet^+^) (left), stage 0/stage 1 iNKT cells (CD44-NK1.1-TCRβ^+^CD1d-Tet^+^) (center) and γδNKT (TCRγδ^+^Vδ6.3^+^) cells (right) is shown (staining for iNKT and γδNKT cells were performed independently). Representative FACS plots from one of three independent experiments are shown. 50,000 sorted CD4^+^CD8^−^ thymocytes from PLZF^+/+^ and PLZF^lu/lu^ Rag1^−/−^Vγ1Vδ6.4 TCR transgenic animals **(C)** or 4000 sorted thymic iNKT cells (TCRβ^+^CD1d-Tet^+^) from wt and PLZF^lu/lu^ mice **(D)** were stimulated with IL12 and IL18 for 48 h or left unstimulated. Production of IFNγ was measured by ELISA. **(E)** CD25 expression by wt and PLZF-deficient cells in response to IL-12 or anti-CD3 treatment.

About 7% of the genes whose expression was affected by PLZF twofold or more are cytokine/chemokine receptors and downstream components of cytokine receptor signaling: *Il18r1, Il8rb, Il12rb, Ccr2, Ccr10, Irak3* are upregulated, while *Stat4, Il4ra, Il6st*, and *Ifngr1* are downregulated by PLZF. This statistically significant (*p* = 2.7 × 10^−5^, DAVID functional annotation tool; Huang et al., 2008) enrichment suggests that PLZF programs NKT cells to respond to a distinct set of extracellular stimuli. IL18R and IL12R signaling was previously implicated in foreign antigen-independent effector responses, where NKT cells were capable of effector cytokine responses without exogenous antigen stimulation. Expression of both IL12Rb1 and IL18R1 on iNKT cells was dramatically decreased in the absence of PLZF – as judged by staining of thymocytes from mixed bm chimeras (Figures 5A,B). Importantly, this difference was obvious as early as stage 0/1. A similar decrease in IL18R1 but not in IL12Rb1 was observed in γδNKT cells (Figures 5A,B).

Although a general defect in IFNγ production by *PLZF*-deficient NKT cells has been reported (Kovalovsky et al., 2008; Savage et al., 2008), in case of stimulation by IL-12 and IL-18 the defect may be compounded by low receptor expression. Consistent with this idea, *PLZF*-deficient cells were incapable of an adequate response to IL-12 and IL-18 cytokines. Wt but not *PLZF^lu/lu^* TCR transgenic γδNKT cells were able to produce a high level of IFNγ in response to a combination of IL-12 and IL-18 (Figure 5C). A similar, though less complete defect was observed in *PLZF^lu/lu^* iNKT cells (Figure 5D). Decreased IFNγ secretion may be not only the result of an intrinsic effect, but also of the dramatically reduced levels of IL-12 and IL-18 receptor expression (Kovalovsky et al., 2008; Savage et al., 2008).

Wt cells also robustly upregulated CD25 in response either to IL-12 or anti-CD3 stimulation. In contrast, *PLZF^lu/lu^* iNKT cells reacted to anti-CD3 but not to IL-12 (Figure 5E), showing that the modulation of *IL12Rb1* expression by PLZF is essential for the responsiveness of iNKT cells to IL-12.

## Discussion

We show here that PLZF, a key transcription factor whose expression is sufficient to confer NKT-like cell surface phenotype, homing pattern, and cytokine secretion profile to conventional T cells, regulates an unexpectedly small set of genes. However, at least 29 out of the 146 genes whose expression changed twofold or more had known immunological functions (DAVID functional annotation tool; Huang et al., 2008). Some of these genes are known to be important for NKT cell development, homeostasis and function – and together explain a part of the PLZF-deficient NKT cell phenotype. Although our expression profiling compared total TCR transgenic *Rag*-deficient γδNKT thymocytes, in order to address the danger that *PLZF*-deficient iNKT cells may be the products of selection – most differences were reproduced in wt NKT cells, at the earliest developmental stage where PLZF is expressed and prior to any possible developmental block due to *PLZF* deficiency.

While rapid production of effector cytokines in response to TCR and cytokine stimulation is a hallmark property of NKT cells, we found that, unexpectedly, at least at steady state, the expression of cytokine genes was not dramatically changed in *PLZF^lu/lu^* thymocytes: *Ifng, Il10, Il4, Il13*, and *Tnf* were regulated less than twofold. The observed changes at the protein level may occur posttranslationally, or may become evident only upon activation of the cell. PLZF-regulated genes in T cells showed no obvious overlap with genes induced by ectopic PLZF expression in a human monocytic cell line (Xu et al., 2009a), suggesting that cell type-specific context can influence the set of PLZF targets.

On the other hand, expression of several cytokine and chemokine receptors, as well as molecules involved in downstream signaling from these receptors were affected – suggesting that PLZF in some cases acts to program the cell’s trafficking pattern and to shape its response to environmental signals, rather than changing the cell intrinsically. Of particular importance are the receptors for IL-12 and IL-18, which in concert mediate a unique part of the NKT cell program – the ability to produce effector cytokines without stimulation with foreign antigen. We show that in the absence of PLZF both receptors are expressed at a lower level at the mRNA (Figure 1A) and at the protein level (Figures 5A,B), and accordingly that the deficient cells produce less IFNγ in response to stimulation with IL-12 and IL-18 in the absence of accessory cells (Figures 5C,D). CD25 upregulation in response to IL-12 was also impaired in *PLZF^lu/lu^* iNKT thymocytes, while they retained the ability to respond to TCR stimulation (Figure 5E).

PLZF also regulates a cluster of genes involved in T cell help to B cells. This cluster includes *ICOS, CD40L, c-Maf* (as shown here) as well as a number of cytokines (Kovalovsky et al., 2008; Savage et al., 2008). Both iNKT (Leadbetter et al., 2008) and γδNKT (Felices et al., 2009) cells can provide help to B cells and induce immunoglobulin class switching; our results suggest that PLZF may regulate this important function.

**Figure A2:**
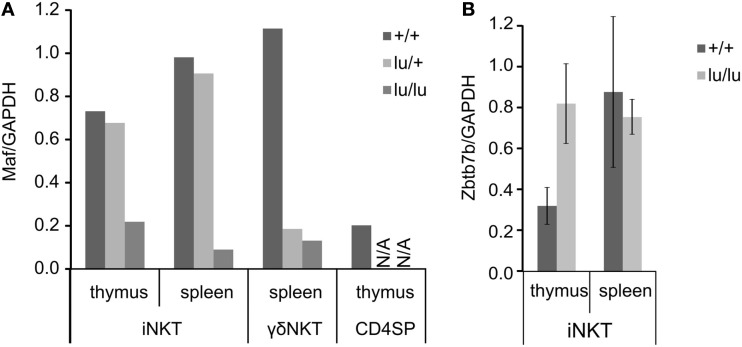
**Expression of Maf (A) and Zbtb7 (B) mRNA analyzed by TaqMan RT-PCR**. Cells from thymi or spleens of mice with the indicated genotype were isolated and stained with PBS-57-loaded CD1d tetramers and antibodies against TCRβ, CD24, CD44 and NK1.1. iNKT, γ δNKT or CD4 single positive were sorted, mRNA was extracted, and subjected to RT-PCR. Error bars represent SD from independent mice.

The transcription factor ThPOK (encoded by *Zbtb7b*) was found among genes regulated by PLZF in TCR transgenic mice (Figure 1A). ThPOK – a BTB-POZ family member that regulates CD4 T cell development – was recently shown to be required for development of iNKT (Engel et al., 2010; Wang et al., 2010) and γδNKT (Alonzo et al., 2009; Park et al., 2010) cells. However, in TCR non-transgenic mice no consistent difference in *ThPOK* expression between *PLZF*^+*/*+^ and *PLZF^lu/lu^* NKT cells was observed (Figure A2B in Appendix), suggesting that regulation of *ThPOK* expression by PLZF does not play a non-redundant role under physiological conditions.

We found evidence for *PLZF* haploinsufficiency. On the level of transcription, for the vast majority of genes either upregulated or downregulated in *PLZF*-deficient cells, heterozygous cells showed an intermediate level of expression (Tables S1 and S2 in Supplementary Material). ICOS expression level was also intermediate in heterozygous cells at the periphery (Figures 4B,D), and in fact within a single animal showed a direct relationship with the level of PLZF expression (Figure 4A), suggesting that PLZF was a limiting factor in the regulation of ICOS expression. Heterozygous animals also showed an intermediate phenotype with respect to NKT cell numbers in the thymus and at the periphery, as well as their maturation state (Figure A1 in Appendix), showing that in this respect PLZF expression is also a limiting factor.

PLZF is expressed by cells that are thought to be selected by agonist ligands (Baldwin et al., 2004; Moran et al., 2011), and can be induced *in vitro* by strong TCR signaling (Kreslavsky et al., 2009). Strong TCR signal is also required for commitment to the γδ T cell lineage. In the absence of strong TCR signal, γδTCR-expressing cells can be diverted to the αβ T cell lineage, as evidenced by their progression to the CD4^+^CD8^+^ stage (Haks et al., 2005; Hayes et al., 2005; Lauritsen et al., 2009). It was thus possible that PLZF might play a role in commitment to the γδ lineage – at least for Vγ1Vδ6.4 T cells. However, the frequency of double positive cells was unchanged in Vγ1Vδ6.4 TCR transgenic mice on a *PLZF*-deficient background, suggesting that PLZF has little or no impact on the αβ versus γδ lineage decision.

The increased percentage of NK1.1^lo^ CD44^lo^ CD1d tetramer-positive cells in the thymi of PLZF-deficient mice has led to a conclusion that PLZF-deficient NKT cells are developmentally blocked at stage 1 of their development (Savage et al., 2008). We note, however, that the CD44/NK1.1 staining pattern in the absence of PLZF appears diffuse, with a significant proportion of NK1.1^+^ CD44^lo^ cells which are not found in wt or heterozygous animals (Figure A1A in Appendix). Such a pattern may indicate a dysregulation of marker expression, rather than a specific block. Corroborating this idea is the presence of a significant number of CD1d tetramer-positive cells at the periphery in PLZF-deficient animals (Figures A1B,C in Appendix, top panels) – if truly blocked, thymocytes should not gain the capacity to leave the thymus. Some cells in the periphery appear mature according to NK1.1/CD44 staining (Figures A1B,C in Appendix, lower panels). Finally, the *PLZF*-deficient cells can produce IFNγ, albeit at a lower level (Kovalovsky et al., 2008; Kreslavsky et al., 2009) – a property normally acquired at stage 2 – indicating that although they may not have passed through all of the appropriate phenotypic stages, they have indeed progressed past stage 1. Thus, our and others’ data may challenge the notion that *PLZF*-deficient iNKT and γδNKT in the periphery are completely naïve – conversely, they may represent cells that were able to partially compensate for the deficiency.

We cannot ascertain whether the genes we describe are regulated by PLZF directly or indirectly. Indeed, the list of genes whose expression is affected by the presence or absence of PLZF includes several encoding known transcription regulators in addition to *Maf* and *Id2* – including *Rora, Lmo4*, and *Npas2*. We note, however, that *Id2* has been shown to be a direct target of PLZF in another setting (Doulatov et al., 2009), and that the strong correlation between the levels of ICOS and PLZF expressed by individual cells may also suggest a direct relationship between the two.

Our data demonstrate that PLZF controls, either directly or indirectly, the expression of a small set of genes, highly enriched for immune function. This includes molecules known to function in NKT cells – *c-Maf, Id2*, and *ICOS*, as well as the proinflammatory cytokine receptors *IL12Rb1* and *IL18R1*. Thus, PLZF orchestrates a part of innate-like phenotype of NKT cells via a set of target genes that together can partially explain the defects in cytokine secretion, survival, co-stimulation and responsiveness to proinflammatory cytokines observed in *PLZF*-deficient NKT cells. The finding that not all of the features of the NKT phenotype can be attributed to PLZF points to the importance of other factors in regulating the unique innate-like properties of and γδNKT and iNKT cells.

## Conflict of Interest Statement

The authors declare that the research was conducted in the absence of any commercial or financial relationships that could be construed as a potential conflict of interest.

## Supplementary Material

The Supplementary Material for this article can be found online at http://www.frontiersin.org/T_Cell_Biology/10.3389/fimmu.2012.00374/abstract
